# Predictive value of label-free surface-enhanced Raman spectroscopy for locally advanced gastric cancer following neoadjuvant chemoimmunotherapy

**DOI:** 10.3389/fimmu.2025.1666860

**Published:** 2025-09-19

**Authors:** Xinran Wang, Bowen Shi, Jinling Jiang, Wenfang Wang, Kangning Wang, Luke Zhang, Fei Yuan, Weiwu Yao, Huan Zhang

**Affiliations:** ^1^ Department of Radiology, Ruijin Hospital, Shanghai Jiao Tong University School of Medicine, Shanghai, China; ^2^ Department of Oncology, Ruijin Hospital, Shanghai Jiao Tong University School of Medicine, Shanghai, China; ^3^ Department of Radiology, Huadong Hospital, Fudan University, Shanghai, China; ^4^ Department of Pathology, Ruijin Hospital, Shanghai Jiao Tong University School of Medicine, Shanghai, China; ^5^ Department of Radiology, Tongren Hospital, Shanghai Jiao Tong University School of Medicine, Shanghai, China; ^6^ College of Health Science and Technology, Shanghai Jiao Tong University School of Medicine, Shanghai, China; ^7^ Shanghai Key Laboratory of Gastric Neoplasms, Shanghai, China

**Keywords:** gastric cancer, neoadjuvant therapy, chemoimmunotherapy, prognosis, Raman spectroscopy

## Abstract

**Background:**

Although neoadjuvant chemoimmunotherapy (NACI) is increasingly applied in clinical settings, its therapeutic efficacy and prognostic significance remain unclear. This study sought to establish a surface-enhanced Raman spectroscopy (SERS)-based approach for assessing treatment efficacy and predicting prognosis in patients with locally advanced gastric cancer (LAGC) undergoing NACI. In addition, the utility of SERS for molecular and pathological profiling was investigated.

**Methods:**

This retrospective study enrolled 31 patients with LAGC treated with anti-PD-1 inhibitors plus chemotherapy before gastrectomy (May 2018–December 2022). A Raman score (RS) was established from SERS spectral features to predict overall survival (OS). The area under the time-dependent receiver operating characteristic curve (AUC), Cox proportional hazards regression, and concordance index (C-index) were used to evaluate model performance. A nomogram combining RS and ypTNM stage was constructed. Kaplan-Meier analysis assessed the risk stratification capacity. Key spectral bands were analyzed for biomarker identification, and machine learning (ML) models were used for histopathological and molecular classification.

**Results:**

A total of 3,670 spectra from 31 patients were analyzed. The RS, based on Raman spectral features, achieved AUCs of 0.854 (1-year OS) and 0.920 (3-year OS). Lower RS correlated with longer OS (p<0.05). RS served as an independent prognostic factor in multivariable analysis. The nomogram incorporating RS and ypTNM improved prediction for 3-year OS (AUC = 0.955) while maintaining 1-year accuracy. Kaplan–Meier analysis confirmed effective risk stratification (P = 0.01). Nine significant Raman bands were linked to nucleotides, collagen, and proteins. ML models achieved >0.85 accuracy in classifying microsatellite instability (MSI), combined positive score (CPS) of programmed cell death ligand-1 (PD-L1), and tumor regression grade (TRG) based on SERS data.

**Conclusions:**

This study demonstrates that label-free SERS can effectively predict prognosis in NACI-treated LAGC patients and shows promise in molecular and pathological profiling, supporting its potential for clinical application.

## 1 Introduction

Gastric cancer (GC) continues to pose a significant global health burden, being the fifth most frequently diagnosed malignancy and the fifth leading cause of cancer-related death worldwide (1). Surgery remains the primary and most effective treatment for localized GC. However, even after radical resection, patients with advanced GC continue to experience poor outcomes, with 5-year overall survival (OS) rates generally below 50% (2). Consequently, neoadjuvant therapy is increasingly used to improve resection rates and prolong survival in patients with locally advanced gastric cancer (LAGC).

Preoperative neoadjuvant chemotherapy has demonstrated the capacity to diminish tumor staging, increase R0 resection rates, and boost overall survival, hence endorsing its incorporation into treatment guidelines for LAGC (3, 4). However, the pathological complete response (pCR) rate achieved by chemotherapy alone remains unsatisfactory (5). Various studies have shown that chemotherapy and immunotherapy have a strong synergistic effect (6–8). Neoadjuvant chemoimmunotherapy (NACI) has increasingly been implemented into clinical practice. However, its therapeutic effect and long-term prognostic value remain uncertain (9–11). Therefore, there is a significant need for precise evaluation of the treatment efficacy and prognosis of GC patients after receiving NACI.

Responses to neoadjuvant therapies have been evaluated from multiple perspectives. The RECIST criteria are widely used for evaluating therapy response based on imaging techniques (12). The existing standards were inadequate for assessing immunotherapy response, leading to the development of new criteria such as irRECIST and iRECIST for improved evaluation of responses to immunotherapeutic interventions (13, 14). However, these evaluations predominantly concentrate on alterations in tumor size, frequently neglecting the changes in the tumor microenvironment post-treatment. Furthermore, they are intricate for implementation in clinical practice. The efficacy of neoadjuvant immunotherapy can be assessed at the pathological level using pCR, major pathological response (MPR), and tumor regression grade (TRG) (15, 16). However, their clinical utility is constrained by the complexity of the detection procedures, as well as the spatial and temporal heterogeneity within tumors (17). The post-neoadjuvant staging system (ypTNM/ypStage) assesses the true extent of disease at surgery and helps predict postoperative survival in GC patients who received preoperative therapy (18). However, the clinical outcome may differ markedly among patients with the same ypStage due to its highly heterogeneous nature (19). Furthermore, although ypStage indicates the extent of tumor infiltration and invasion, it does not provide information on molecular markers unless integrated with additional diagnostic and laboratory techniques such as immunohistochemistry (IHC), flow cytometric analysis, next-generation sequencing and polymerase chain reaction (20, 21), which are time-consuming, labor-intensive, and expensive. Thus, there is a persistent necessity to establish a more precise, simple, and comprehensive approach for clinically assessing the treatment efficacy and predicting the prognosis of GC treated with NACI.

Raman spectroscopy is a non-destructive, label-free technique that identifies molecule vibrational modes by analyzing inelastically scattered light, hence offering comprehensive molecular fingerprints of samples (22, 23). The enhancement factors of SERS are generally in the range of 10^4^–10^6^, and values as high as 10^8^–10^14^ have been documented under certain conditions (24–26). SERS has been extensively used in many cancers, facilitating the ultrasensitive detection of diverse biomolecular targets, including nucleic acids (DNA, RNA), proteins, metabolites, and extracellular vesicles (27, 28). Recent studies have demonstrated the utility of label-free SERS in cancer diagnosis and tumor biomarker analysis. For instance, Gao et al. combined label-free SERS with a CNN model, achieving rapid and accurate diagnosis of thyroid cancer (23). Chen et al. proposed a novel multitask deep learning framework, ASFN, which integrates label-free SERS with deep learning to enable high-precision detection and quantitative analysis of subtle tumor biomarkers within complex matrices (29). Liu et al. developed a machine learning-assisted SERS requiring minimal blood, delivering stable and accurate classification for rapid, reliable early lung-cancer detection and monitoring (30). However, these investigations have predominantly focused on blood or puncture fluids, with little research addressing tissue sections. The formalin-fixed paraffin-embedded (FFPE) GC tissues retain comprehensive information on tumor characteristics and treatment-induced alterations, thereby serving as ideal specimens for SERS analysis. As previously reported, Raman spectroscopy has been validated as a method for predicting the prognosis of GC through the detection of tissue sections (31). However, the potential of SERS in analyzing postoperative pathological tissues following NACI warrants further investigation.

This study aimed to develop a simple, effective, and comprehensive approach for assessment of therapeutic effectiveness and prognostic prediction of NACI in LAGC using SERS features derived from 31 patients. Furthermore, we investigated the biological relevance of the SERS spectral features and explored its potential for classifying histopathological and molecular characteristics.

## 2 Materials and methods

### 2.1 Patients

**Figure 1:**
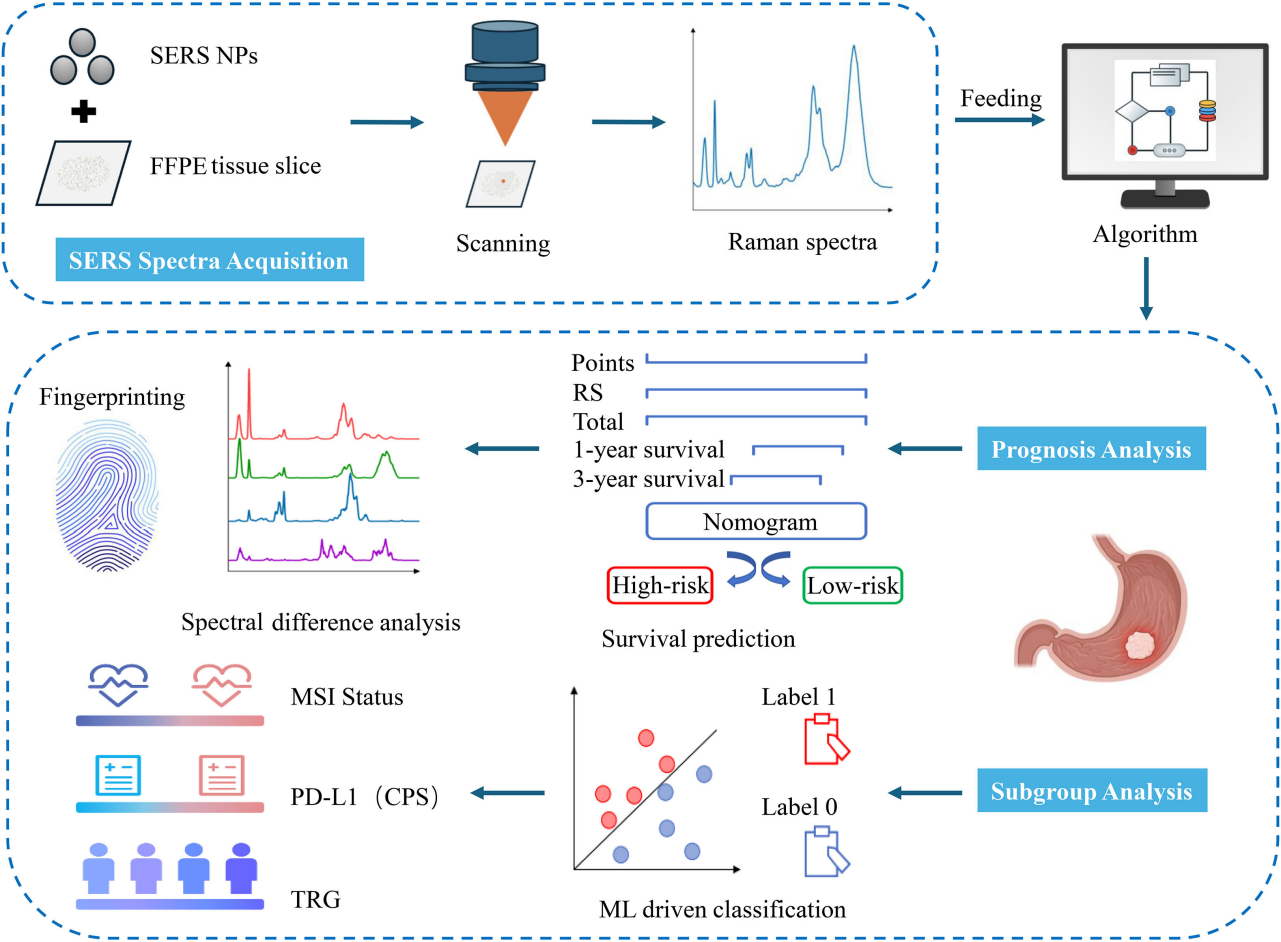
Workflow of SERS for prognosis and subgroup analysis of LAGC patients. SERS spectra were collected from GC tissues and fed into in-house developed algorithms for the construction of a prognosis prediction model. Based on the nomogram model, these sample spectra were divided into high-risk and low-risk groups. Then, the differential spectral features between the groups were analyzed. Additionally, ML classification models were established to differentiate among various pathological subtypes of GC tissues.

**Figure 2:**
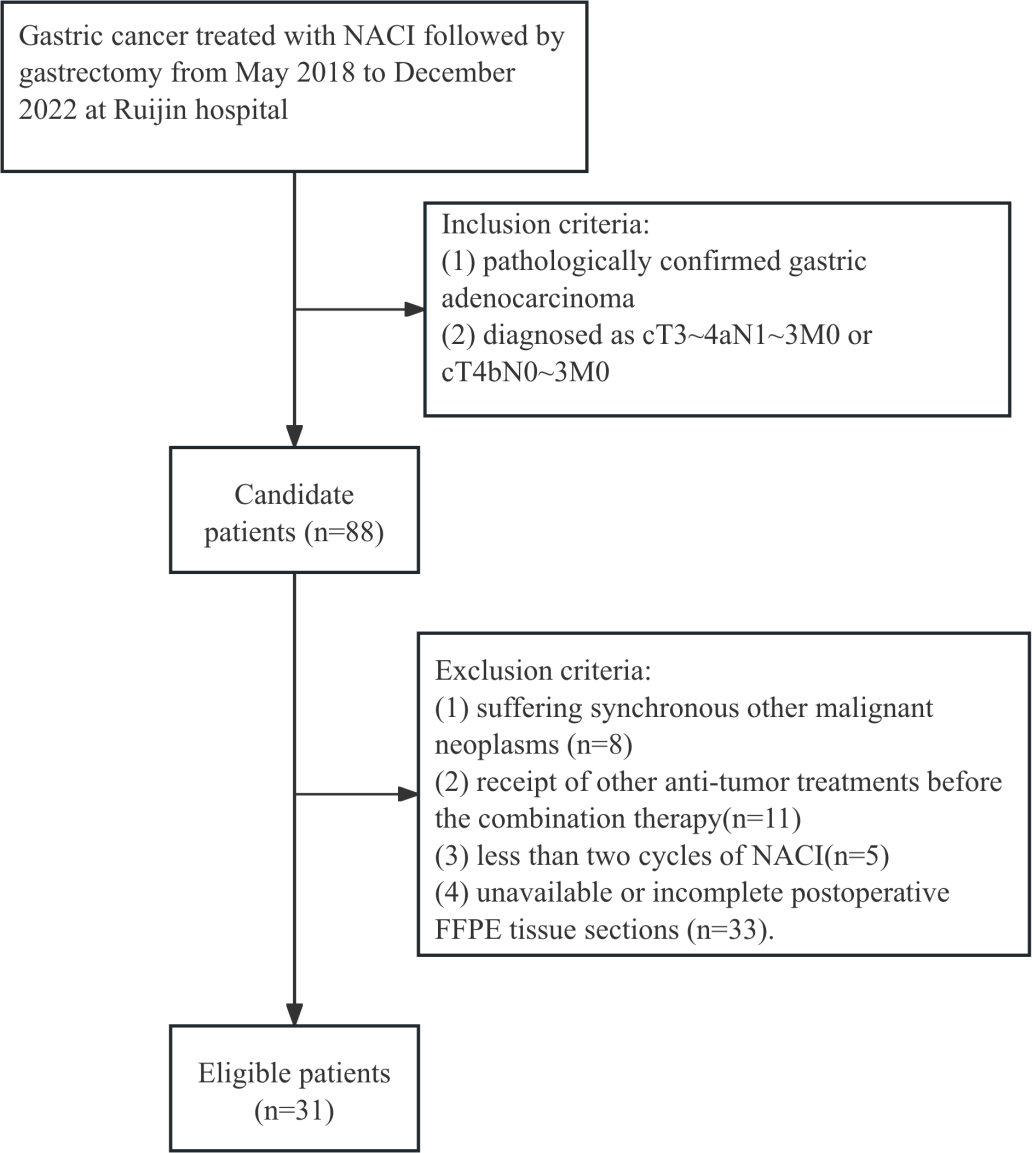
Flowcharts of the patient recruitment process.

The study design is illustrated in **Figure 1**. This retrospective analysis encompassed 31 patients with LAGC who received anti-PD-1 drugs in conjunction with chemotherapy before gastrectomy from May 2018 to December 2022 at Ruijin Hospital, Shanghai, China. All enrolled patients fulfilled these inclusion criteria: (1) pathologically confirmed gastric adenocarcinoma; (2) diagnosed as cT3-4aN1-3M0 or cT4bN0-3M0. The exclusion criteria were (1) suffering synchronous other malignant neoplasms, (2) receipt of other anti-tumor treatments before the combination therapy, (3) less than two cycles of NACI, and (4) unavailable or incomplete postoperative FFPE tissue sections. A patient selection flowchart is shown in **Figure 2**. Demographic and clinicopathological data were retrieved from medical records, including age, sex, the ypTNM stage, MSI status, PD-L1 status, TRG, and follow-up data of 1-year and 3-year OS (**Table 1**). Ethical approvals were obtained from the Ruijin Hospital Ethics Committee, and informed consent was exempted given the retrospective nature of the study.

**Table 1:** The demographics and clinicopathological information of 31 patients.

Variable	Classification	Statistical data
Age (year)		61(23-75)
Gender	male	23
	female	8
Staging	I	5
	II	10
	III	8
	IV	8
MSI status	MSI-H/MMR deficient	6
	MSS/MMR proficient	25
PD-L1(CPS)	≥5	19
	<5	8
	Unknown	4
TRG	0-1	7
	2-3	24
OS (month)		36.5(6.1-82.8)

CPS, combined positive score.

### 2.2 Label-free SERS measurement

The pretreatment of tissue sections and the synthesis of SERS nanoparticles are elaborated in **Supplementary Material**. The SERS spectra were acquired using a fiber optic spectrometer (NOVA2S high sensitivity spectrometer, Ideaoptics, China) equipped with a 20× objective lens and a 1200 lines/mm grating. The excitation wavelength was 633 nm, and each SERS spectrum was acquired with a one-second integration time. The measured wavenumbers spanned from 600 to 1800 cm^−1^ with a spectral resolution of above 1 cm^-1^. Three separate scanning zones (central and peripheral regions along a meridian) were systematically analyzed for each tissue region attached with silver nanoparticles (Ag NPs) to mitigate evaporation-induced coffee ring artifacts and measurement variability. We collected approximately 120 spectra for each sample, with a 10 µm step size. A total of 3,670 Raman spectra were ultimately acquired. The preprocessing processes for spectral data are outlined in the supplementary data, and the average spectrum is illustrated in **Supplementary Figure S1**. Representative spectra of each preprocessing step are shown in **Supplementary Figure S2**.

### 2.3 Raman spectral feature extraction

To tackle the high-dimensionality problem inherent in Raman spectroscopy data, Partial Least Squares Regression (PLSR) was utilized for dimensionality reduction to identify outcome-associated significant spectral features. Five-fold cross-validation was performed to determine the optimal number of Raman spectral features. Finally, two Raman spectral features were selected, and the Raman score (RS) was constructed based on the Cox regression coefficients of each Raman spectral feature. A detailed explanation of the construction of RS has been supplemented in the supporting information. The selected features for the construction of RS are detailed in **Supplementary Table S1** and **Supplementary Figure S7**.

### 2.4 Nomogram construction

The prognostic value of the RS and clinicopathological factors was assessed using univariate Cox regression. Significant variables (p < 0.05) were then entered into multivariate analysis. Hazard ratios were presented alongside their respective 95% confidence intervals (CIs), with statistical significance established at a p-value < 0.05. Based on the results of the above analysis, selected variables were integrated into the nomogram to predict the probability of 1-year and 3-year OS rates in LAGC patients following NACI combined with gastrectomy.

### 2.5 Performance evaluation

Time-dependent receiver operating characteristic (ROC) curve analysis was employed to evaluate the prognostic effectiveness of the RS and clinical characteristics. The predictive performance of the RS and clinical factors was evaluated by comparing the area under the time-dependent receiver operating characteristic curves (AUCs) for 1-year and 3-year OS. Harrell’s concordance index (C-index) was used to assess improvements in the predictive accuracy of the prognostic nomogram. Calibration curves were utilized to evaluate the alignment between expected probabilities and actual outcomes of the nomogram. Patients were grouped into high- and low-risk categories using the median nomogram score as the cutoff. The Kaplan-Meier analysis, together with the log-rank test, was utilized to evaluate OS among the designated risk categories, thereby establishing the prognostic relevance of the nomogram.

### 2.6 Statistical analysis

Prominent Raman spectral bands distinguishing high-risk from low-risk groups were preliminarily identified by one-way analysis of variance (one-way ANOVA), accompanied by an assessment of effect size (η²). Subsequently, neighboring peaks were consolidated utilizing the DBSCAN (Density-Based Spatial Clustering of Applications with Noise) algorithm, and intergroup variations were confirmed through Tukey’s HSD test. Peak intensity differences were determined using localized peak extraction, normality assessment (Shapiro-Wilk, α=0.05), and suitable statistical analyses (t-test or Mann–Whitney U test), subsequently accompanied by Bonferroni-corrected violin plots for significance visualization. All statistical analyses were performed using Python version 3.8.1 (www.python.org) and R version 3.6.1 (www.r-project.org). Unless otherwise specified, all tests were two-sided, with a significance threshold of P < 0.05.

## 3 Results

### 3.1 Clinicopathological and Raman characteristics

The study cohort comprised 31 subjects (23 males [74.2%], 8 females [25.8%]) with a median age of 61 years (range 23-75). Based on the ypTNM staging criteria, the cohort was distributed as follows: stage I (n=5, 16.1%), stage II (n=10, 32.3%), stage III (n=8, 25.8%), and stage IV (n=8, 25.8%). Baseline information for all 31 patients is summarized in **Table 1**. Two Raman spectral features, PLS1 and PLS2, were identified and used to formulate the RS, which was calculated as a weighted combination based on Cox regression coefficients. The Cox proportional hazards regression model was utilized to evaluate these factors and identify potential independent predictors of OS.

### 3.2 Prognostic analysis by label-free SERS

As shown in **Table 2**, univariate analysis identified only the RS (HR = 2.718; 95% CI: 1.516–4.872; P < 0.001) and ypTNM classification (HR = 8.204; 95% CI: 1.020–65.983; P = 0.048) as significant predictors of overall survival. HR quantifies the effectiveness of treatment (32). An HR exceeding 1 denotes poorer overall survival, whereas patients with lower risk scores generally show improved survival outcomes. Although the HR for ypTNM classification was 8.204, with a broad 95% confidence interval (95% CI, 1.020–65.983), suggesting some uncertainty in the estimate, the lower bound still exceeds 1, indicating statistical significance. Moreover, the calculated C-index of 0.699 (0.550–0.847) reflects a fair discriminative ability for survival prediction, supporting ypTNM classification as a key prognostic factor. In the multivariate analysis (**Table 3**), we compare the RS with the ypTNM classification. The results indicate that the RS is an independent prognostic factor of OS (HR = 2.415; 95% CI: 1.337–4.363; P = 0.003), whereas ypTNM classification shows no significant association (HR = 3.291; 95% CI: 0.366–29.601; P = 0.288).

**Table 2:** Univariate analysis of variables associated with overall survival.

Variable	Univariable	P Value	C-index (95% CI)
	HR(95%CI)		
Age	1.144(0.305-4.293)	0.842	0.488(0.312-0.664)
Gender	2.946(0.368-23.609)	0.309	0.563(0.413-0.713)
ypTNM class	8.204(1.020-65.983)	0.048	0.699(0.550-0.847)
RS	2.718(1.516-4.872)	<0.001	0.874(0.774-0.973)

**Table 3:** Multivariate analysis of variables associated with overall survival.

Variable	Multivariable	P value	C-index
	HR (95%CI)		
ypTNM class	3.291(0.366-29.601)	0.288	0.888(0.799-0.977)
RS	2.415(1.337-4.363)	0.003	

**Figure 3:**
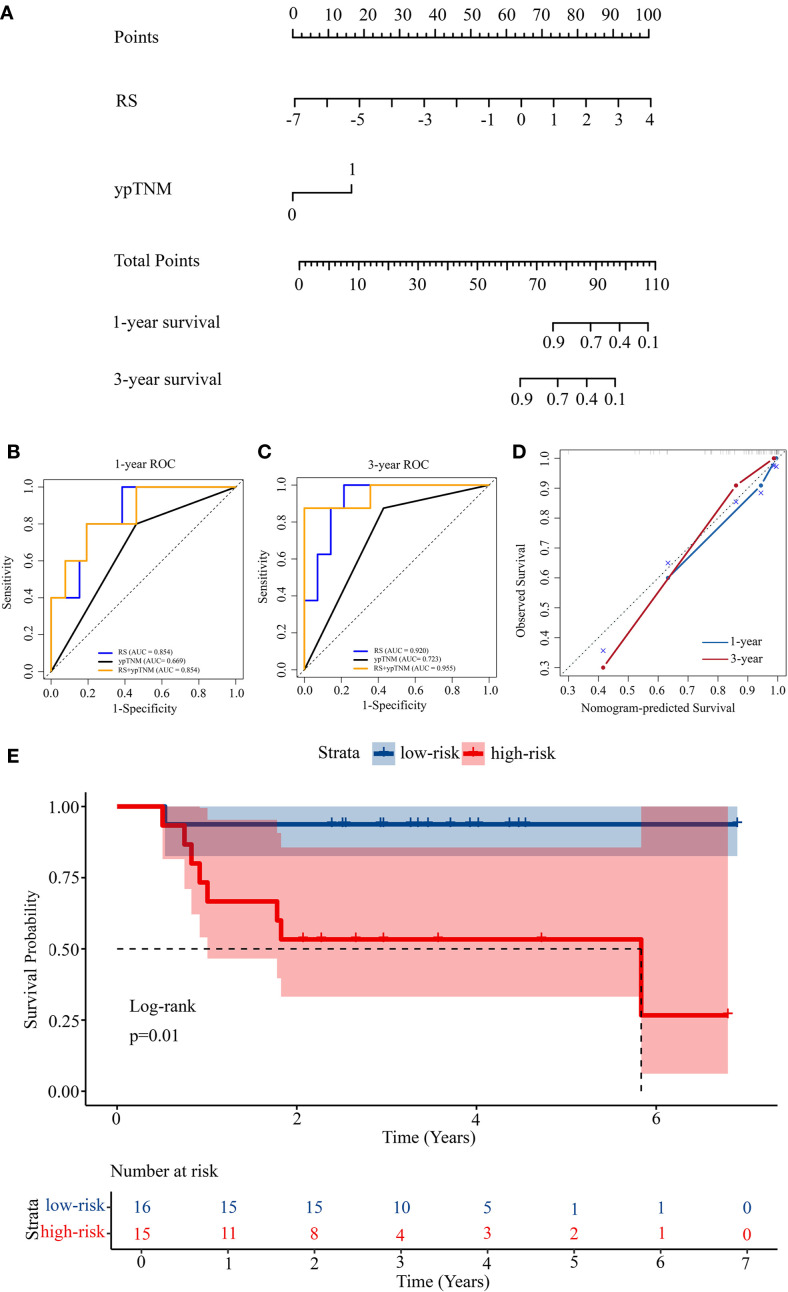
Construction of a prognosis prediction model. **(A)** Nomogram for 1-year and 3-year OS based on the RS and ypTNM. **(B)** 1-year ROC curves of the RS, ypTNM, and their combined model. **(C)** 3-year ROC curves of the RS, ypTNM, and their combined model. **(D)** Calibration curves of the nomogram in terms of agreement between predicted and observed 1-year and 3-year OS. **(E)** Kaplan-­Meier survival analysis of OS in the high-risk and low-risk groups.

Given the therapeutic relevance of the ypTNM classification as published by the AJCC (18), we integrated both RS and ypTNM classification to develop a nomogram for predicting OS in LAGC patients undergoing NACI, as illustrated in **Figure 3A**. The nomogram correlates the cumulative score from all prognostic indicators with the anticipated survival probability, where elevated scores signify a diminished chance of patient survival. **Figure 3A** illustrates the points allocated for the RS and ypTNM methods, which are automatically determined by the algorithm based on effect size: RS receives 100 points, while the ypTNM classification is assigned only 18 points, signifying that RS plays a predominant role in the predictive analysis.

### 3.3 Performance and validation of prognostic model

The nomogram exhibited strong discriminative capability, achieving a C-index of 0.888 (95% CI: 0.799-0.977), as presented in **Table 3**. We constructed ROC curves to further examine the discriminative performance of our model. **Figure 3B** illustrates that the 1-year ROC curves indicate the RS prediction model (AUC = 0.854) outperforms the ypTNM class model (AUC = 0.669). The integration of RS with the ypTNM category (AUC = 0.854) does not yield any enhancements, suggesting that the RS approach is sufficiently effective alone. In the 3-year ROC curves (**Figure 3C**), the RS model demonstrates superior performance (AUC = 0.920) compared to the ypTNM class (AUC = 0.723), while the combined model achieves the highest results (AUC = 0.955), suggesting that the ypTNM class also enhances predictive accuracy. The calibration curves of the nomogram in **Figure 3D** for OS demonstrated strong concordance between the estimated and actual observations. The results demonstrate that the nomogram can provide individualized survival predictions for LAGC patients undergoing NACI. The RS method shows potential as an innovative clinical tool for enhancing prognostic prediction.

Using the median nomogram score as a cutoff, patients were divided into high-risk and low-risk cohorts. The median OS for the high-risk cohort was 2.57 years (range: 0.75–5.83), whereas for the low-risk cohort, it was 3.49 years (range: 0.53–6.90). The survival differences between high-risk and low-risk groups were assessed using Kaplan–Meier analysis, subsequently followed by a log-rank test to evaluate statistical significance (**Figure 3E**). The observed difference in survival distributions demonstrated statistical significance (P = 0.01), underscoring the prognostic relevance of the risk stratification.

### 3.4 Illustration of significant differential Raman bands

**Figure 4:**
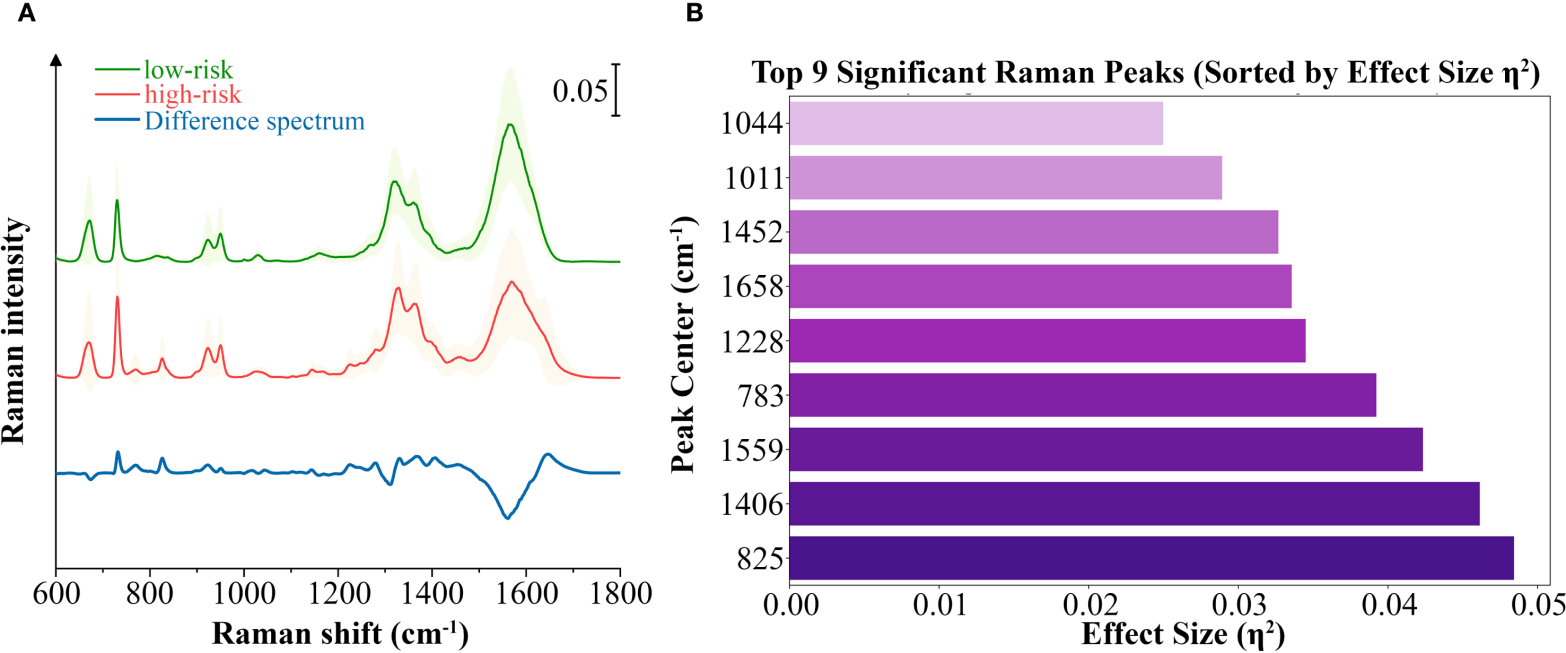
Analysis of the differential spectral features between the high-risk and low-risk groups. **(A)** Averaged SERS spectra with standard deviations of high-risk group and low-risk group samples, as well as the averaged difference spectrum (high–low). Standard deviation values are shown in shadow. **(B)** The top 9 significant differential bands sorted by effect size η².

**Figure 5:**
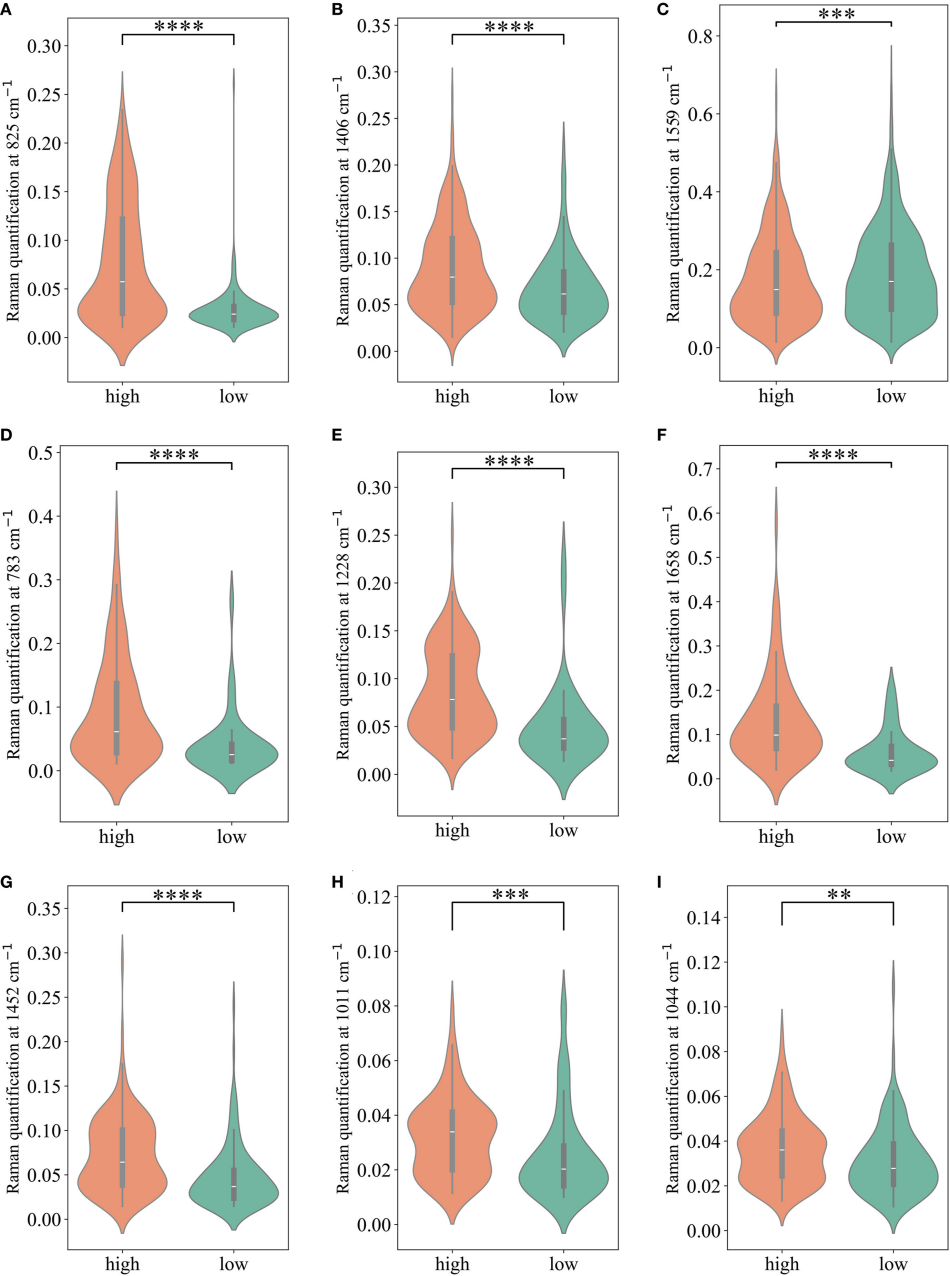
The comparison of Raman peak intensities for each of the significant Raman bands. **(A-I)** present comparisons of Raman peaks at 825 cm^-1^, 1406 cm^-1^, 1559cm^-1^, 783cm^-1^, 1228cm^-1^, 1658cm^-1^, 1452cm^-1^, 1011cm^-1^,1044cm^-1^, respectively. * P < 0.05; ** P < 0.01; *** P < 0.001; **** P < 0.0001.

We calculated the average Raman spectra for the high-risk and low-risk groups, respectively (**Figure 4A**). Although the mean spectra show distinct differences between the two groups, the averaging process may obscure spectral variations present at different locations within individual samples. Therefore, we further analyzed the most significant differential bands between the two groups. Nine significantly different bands were ultimately detected (**Figure 4B**). The effect size (η²) reflects the magnitude of the actual difference in spectral intensities between the two groups. Based on the ranking of effect sizes, the differential Raman spectra were ordered as follows: 825 cm^-1^, 1406 cm^-1^, 1559 cm^-1^, 783 cm^-1^, 1228 cm^-1^, 1658 cm^-1^, 1452 cm^-1^, 1011 cm^-1^, and 1044 cm^-1^. To further validate these findings, we tested the statistical differences in peak intensities at the characteristic wavenumbers and displayed the outcomes as violin plots (**Figures 5A–I**). SERS is intrinsically surface-specific, with the recorded spectra reflecting molecules adsorbed on plasmonic nanostructures (33, 34). In this study, we conducted a comparative rather than quantitative analysis. Under strictly matched acquisition and preprocessing conditions, between-group differences in SERS intensities indirectly reflect variations in the surface-accessible molecular fingerprints of the tissue sections. The violin plots clearly reveal significant intensity differences in the nine Raman bands between the high-risk and low-risk groups. The primary SERS bands and their corresponding biochemical attributions are detailed in **Table 4**. As shown in the violin plots, the Raman peaks at 1044 cm^−1^, 1406 cm^−1^, and 1658 cm^−1^, linked with collagen, were elevated in the high-risk group compared to the low-risk group, signifying increased collagen content (1044 cm^−1^ pertains to proline, 1406 cm^−1^ relates to the bending modes of methyl groups, and 1658 cm^−1^ corresponds to Amide I). The intensity of the Raman peaks at 1228 cm^−1^, 1452 cm^−1^, and 1011 cm^−1^, associated with proteins, showed a marked increase in the high-risk group relative to the low-risk group (1228 cm^−1^ corresponds to Amide III, 1452 cm^−1^ pertains to δ(CH2) (CH3), and 1011 cm^−1^ is linked to phenylalanine). In contrast, the Raman signal at 1559 cm^−1^ associated with Tryptophan exhibited a notable decrease in the high-risk group versus the low-risk group. It suggested a potential disparity in protein structure between the two groups, even post-neoadjuvant treatment.

**Table 4:** Characteristic Raman bands between high-risk and low-risk groups, and their assignment to biochemical components.

Peak center (cm^−1^)	Feature(cm^−1^)	Assignment	Main molecules	P value
1044	1043	Proline	Collagen (51)	p<0.01
1011	1004	Phenylalanine	Protein, collagen (51)	p<0.001
1452	1449	*δ*(CH2)(CH3)	Protein, lipid (52)	p<0.0001
1658	1655	Amide I	Collagen, protein (51)	p<0.0001
1228	1224	Amide III (*β* sheet structure)	Protein (51)	p<0.0001
783	782	DNA	Nucleic acid (51)	p<0.0001
1559	1555	Tryptophan	Protein (53)	p<0.001
1406	1401	Bending modes of methyl groups	Collagen (51)	p<0.0001
825	826	O-P-O stretch DNA	Nucleic acid (51)	p<0.0001

### 3.5 Classification of histopathological and molecular subtypes by SERS spectra

To further examine the effectiveness of SERS in differentiating various histopathological and molecular features of LAGC after NACI, we performed subgroup analyses based on MSI status, PD-L1 status, and TRG. The enrolled patients were categorized into subgroups: MSI-H and MSS (3670 spectra), PD-L1 (CPS≥5) and PD-L1 (CPS<5) (3015 spectra), and TRG 0–1 and TRG 2-3 (3670 spectra). We assessed the diagnostic performance of four machine learning models: Principal Component Analysis–Linear Discriminant Analysis (PCA-LDA), Random Forest (RF), Support Vector Machine (SVM), and Convolutional Neural Network (CNN). The construction process of these models is elaborated in **Supplementary Material**. In the MSI-H and MSS subgroups, RF and CNN achieved high accuracies of 0.9183 and 0.9128, respectively, while SVM and PCA-LDA achieved accuracies of 0.8978 and 0.8624, respectively. In the PD-L1 (CPS≥5) and PD-L1 (CPS<5) subgroups, RF and CNN also achieved high accuracies of 0.9083 and 0.9104, respectively, while SVM and PCA-LDA achieved accuracies of 0.8905 and 0.8624, respectively. In the TRG 0–1 and TRG 2–3 subgroups, the CNN model achieved the highest accuracy of 0.8719; the accuracy of RF and SVM followed closely at 0.8692, while PCA-LDA had the lowest accuracy, still reaching 0.8188. ROC curve analysis for MSI subgroups showed AUC values of 0.92 (PCA-LDA), 0.97 (RF), 0.96 (SVM), and 0.97 (CNN). AUC values approaching 1 indicate superior algorithm performance. This verifies that within the MSI status subgroup, all four models demonstrated strong classification efficacy. In the PD-L1 status subgroups, AUCs of 0.89, 0.95, 0.94, and 0.95 were achieved by the PCA-LDA, RF, SVM, and CNN models, respectively. Except for the marginally lower performance of PCA-LDA, RF, SVM, and CNN all showed notably better classification performance for the MSI status subgroup. ROC analysis of the TRG subgroups yielded AUC values of 0.77 (PCA-LDA), 0.88 (RF), 0.88 (SVM), and 0.88 (CNN). RF, SVM, and CNN models demonstrated equivalent classification performance, whereas PCA-LDA remained inferior to the other three models. The performance metrics for the four classification models across subgroups are summarized in **Table 5**, including accuracy, sensitivity, specificity, and AUC. The ROC curves and confusion matrices are depicted in **Supplementary Figures S3**-**6**.

**Table 5:** Performance comparison of four ML models across subgroups (accuracy, sensitivity, specificity, and AUC).

Subgroup	Accuracy	Sensitivity	Specificity	AUC
MSI Status				
PCA-LDA	0.8624	0.9525	0.6346	0.92
RF	0.9183	0.9848	0.7500	0.97
SVM	0.8978	0.9829	0.6827	0.96
CNN	0.9128	0.9525	0.8125	0.97
PD-L1 (CPS)				
PCA-LDA	0.8624	0.9816	0.5536	0.89
RF	0.9038	0.9885	0.6845	0.95
SVM	0.8905	0.9908	0.6310	0.94
CNN	0.9104	0.9793	0.7321	0.95
TRG				
PCA-LDA	0.8188	0.2789	0.9540	0.77
RF	0.8692	0.3741	0.9932	0.88
SVM	0.8692	0.3673	0.9949	0.88
CNN	0.8719	0.6122	0.9370	0.88

## 4 Discussion

In this study, we established a predictive model for LAGC patients receiving NACI by integrating the PLSR approach with SERS and clinical ypTNM staging, resulting in accurate survival outcome predictions. We effectively categorized the patients into high-risk and low-risk cohorts and examined the disparities in molecular signatures of biological constituents between the two groups. We further employed four different machine learning models to classify the histopathological and biomolecular features of LAGC based on SERS spectral characteristics, each with an accuracy over 0.85.

Our research demonstrates that label-free SERS can accurately predict the prognosis of LAGC patients receiving NACI. We innovatively retrieved Raman spectral features to develop a Raman score for nomogram construction, achieving AUCs of 0.854 and 0.920 for 1-year and 3-year survival, respectively, surpassing the ypTNM stage, which had AUCs of 0.669 and 0.723 for 1-year and 3-year survival, respectively. Their integrated model attained an AUC of 0.854 for 1-year survival, matching that of the RS alone, suggesting that the ypTNM staging did not enhance the combined model. For 3-year survival, the combined model attained an AUC of 0.955, indicating that while the ypTNM staging offered supplementary value, the RS remained the principal factor influencing the efficacy of the nomogram model. Previous studies have also extensively investigated various biomarkers to predict immunotherapy response and prognosis in GC, such as PD-L1 expression (35), tumor mutational burden (36), MSI status (37), Epstein-Barr virus infection (38), circulating tumor DNA (ctDNA) (35), gut microbiota (38), Peripheral Blood Biomarkers (38), Gene Expression Profile (38) and radiomics-derived signatures (39). Nonetheless, these biomarkers offer merely a unidimensional view of the tumor, overlooking the diverse and dynamic alterations induced by NACI. In contrast, SERS enables the detection of molecular-level chemical bond information within tissues, offering insights into both composition and metabolic activity. Its simplicity and rapid operation, combined with the capacity to extract multiple dimensions of tumor-related information in a single scan, render it a superior alternative to conventional histopathology to a certain degree.

Our study highlights that label-free SERS can sensitively detect subtle biochemical remodeling in GC tissues following NACI, especially in signals related to nucleotides and collagen. The molecular alterations, which have been minimally investigated in previous research, highlight the distinctive diagnostic significance of SERS beyond traditional evaluation techniques. A comparative examination of the Raman spectra of high-risk and low-risk groups was done, finding considerable differences, especially in bands associated with nucleotide metabolites, collagen, and proteins. These data indicate distinct biochemical profiles and protein conformations between the two groups. The augmented intensity of nucleotide-associated Raman bands in the high-risk group indicates enhanced nucleic acid metabolism, potentially reflecting greater proliferative activity following neoadjuvant therapy. Furthermore, changes in collagen-associated bands may signify extracellular matrix remodeling, a vital element affecting tumor invasiveness and treatment efficacy.

We found that peaks at 783 cm^−1^ and 825 cm^−1^ were significantly elevated in the high-risk group following neoadjuvant therapy compared to the low-risk group, indicating a higher relative contribution of nucleic-acid related bands in the high-risk group. Previous studies have indicated that a decrease in ctDNA levels during immunotherapy frequently forecasts positive treatment responses and extended OS (40–42). The unfavorable survival outcome in the high-risk group may suggest a greater tumor load and ctDNA burden. We also noted that Raman peaks at 1406 cm^-1^, 1044 cm^−1^ and 1658 cm^−1^ associated with collagen, were elevated in the high-risk group compared to the low-risk group. The extracellular matrix (ECM) significantly impacts tumor development and immune evasion in GC. The overexpression of components like collagen, laminin, and fibronectin results in heightened matrix stiffness, impeding T-cell penetration and disrupting integrin signaling (43). Additionally, the ECM sequesters immunosuppressive molecules like TGF-β (44). Cancer-associated fibroblasts further remodel the ECM by depositing matrix proteins and enhancing collagen cross-linking, reinforcing an immunosuppressive microenvironment (45). Therefore, we speculate that enhanced collagen related SERS intensities in the high-risk group indicate a denser or stiffer extracellular matrix that may impair the penetration and efficacy of immunotherapeutic agents, thereby contributing to poorer prognostic outcomes. These findings underscore the potential role of collagen-mediated matrix remodeling as a critical barrier to effective immunotherapy in GC.

Furthermore, our study also classified the histopathological and molecular characteristics of LAGC in SERS based on four different machine learning models: PCA-LDA, RF, SVM and CNN. In the PD-L1 and MSI status subgroups, all four models achieved an accuracy of more than 0.85, with AUCs surpassing 0.89. In the TRG subgroup, all four models achieved an accuracy of more than 0.8, with AUCs surpassing 0.77. While the sensitivity of these models for classifying TRG was not satisfactory, we speculate that this is due to sample imbalance, which may have caused a partial loss of positive identification rate during the machine learning process. Prior research on Raman spectroscopy in GC has predominantly concentrated on oncological diagnostics (46–49). This study focused on variables such as PD-L1, MSI, and TRG, which past research has shown as major prognostic factors (36, 50). The diagnosis of these indications predominantly depends on pathological staining and IHC, which are both time-consuming and labor-intensive processes. This work shows that spectral data from a single SERS scan may effectively classify and diagnose various histopathological and biomolecular markers using conventional machine learning techniques.

Although our study yielded promising results, its small sample size and single-center design limit the generalizability of the findings. Future studies with larger, multicenter cohorts are warranted to further validate these results. The retrospective aspect of this investigation presents inherent limitations. Furthermore, the continued advancement of the SERS-based tissue measurement platform is crucial for accurate prognosis prediction and biomarker identification in LAGC patients undergoing NACI, thus facilitating more informed precision medicine.

## 5 Conclusion

This study demonstrates not only the ability of label-free SERS to precisely predict prognosis in LAGC following NACI, but also the potential of SERS in biomolecular and histopathological analysis. SERS provides efficient and comprehensive analytical capabilities. Beyond GC, the adaptability of this technique can also be applied to other diseases, highlighting its extensive translational potential.

## Data Availability

The original contributions presented in the study are included in the article/**Supplementary Material**. Further inquiries can be directed to the corresponding authors.
