# Supplementary material for: Predictive value of label-free surface-enhanced Raman spectroscopy for locally advanced gastric cancer following neoadjuvant chemoimmunotherapy
Source: Front Immunol. 2025 Sep 19;16:1666860. doi: 10.3389/fimmu.2025.1666860 (PMC12491268; doi:10.3389/fimmu.2025.1666860)
Supplement: Supplementary file 1 [file DataSheet1.pdf]

Preparation of materials

(1) Pre-treatment of tissue sections

All tissue specimens were obtained post-surgery and subsequently embedded in paraffin. Each tissue was serially sectioned into two 8-μm slices, which were nearly identical: one was used for preparing FFPE sections, and the other for H&E staining. The FFPE slices were dewaxed according to a standard clinical dewaxing protocol before Raman measurements.[1] In brief, slides were sequentia lly immersed and gently agitated for 1 minute in each of the following: 2 rou nds of xylene substitute baths (Aladdin, Shanghai, China), 3 rounds of 100 % ethanol baths, and 3 rounds of ultrapure water baths. Finally, the slides were d ried in a vacuum dryer for 20 min at 37 ℃.

(2) Preparation of Ag NPs

Citrate-reduced silver (Ag) nanoparticles were synthesized using a modified procedure based on the approach outlined by Lee and Meisel,[2] as detailed in a prior publication.[3] In summary, 9.2 mg of $AgNO_3$ was solubilized in 75 mL of ultrapure water and heated to boiling while being stirred continuously. Subsequently, 2 mL of sodium citrate solution (10.1 mg/mL) was administered dropwise. The liquid was kept boiling for a further hour in darkness, then cooled to room temperature with vigorous stirring. The resultant colloid was preserved at 4 °C and shielded from light until subsequent utilization. Before SERS experiments, the silver colloid solution underwent 2 minutes of ultrasonication to achieve uniform dispersion of the silver nanoparticles. A 5 μL aliquot of silver colloid was deposited onto the surface of dewaxed and rehydrated gastric tissue sections using a calibrated micropipette, followed by a 15-minute incubation before spectrum capture.

Raman data preprocessing

Raman spectrum data were preprocessed using LabSpec 6_6_1 software. Cosmic rays were initially eliminated, followed by noise reduction achieved through smoothing with a Savitzky-Golay filter. Raman spectra were refined using a Savitzky-Golay filter with a polynomial degree of 3 and a window size of 15 data points. Baseline correction was performed using the adaptive iteratively reweighted penalized least squares (airPLS) algorithm, with a polynomial order of 3 and a smoothing value (λ) of 150. The spectral data were then standardized through area normalization. The averaged spectrum following preprocessing is depicted in Figure S1. The spectra of each preprocessing step are depicted in Figure S2.

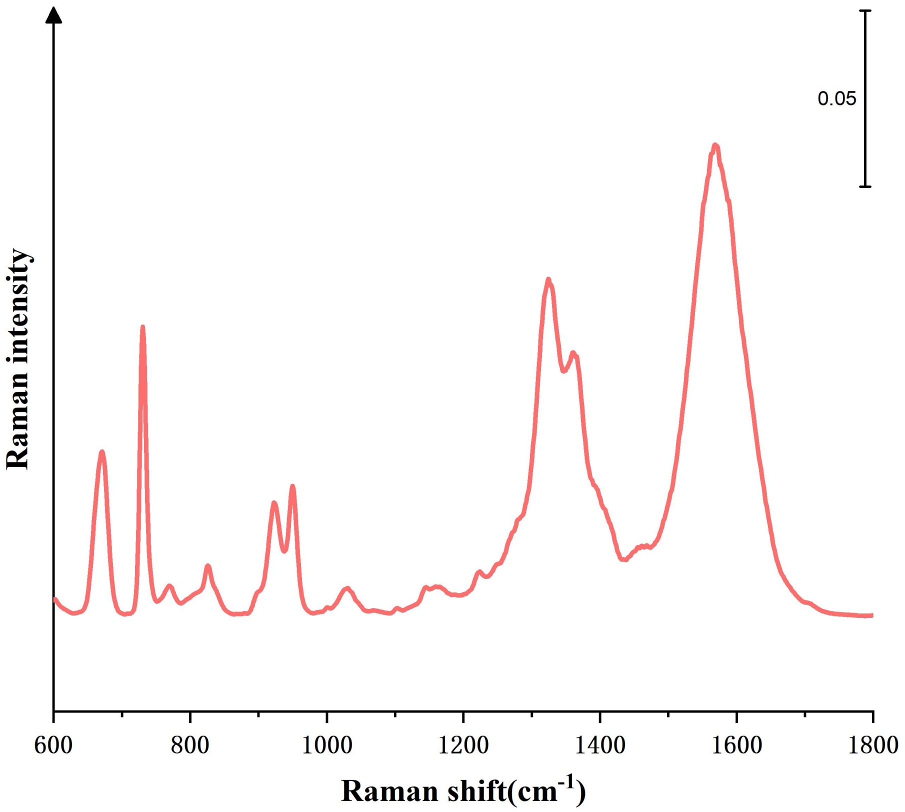

Figure S1. Averaged SERS spectrum of all GC samples.

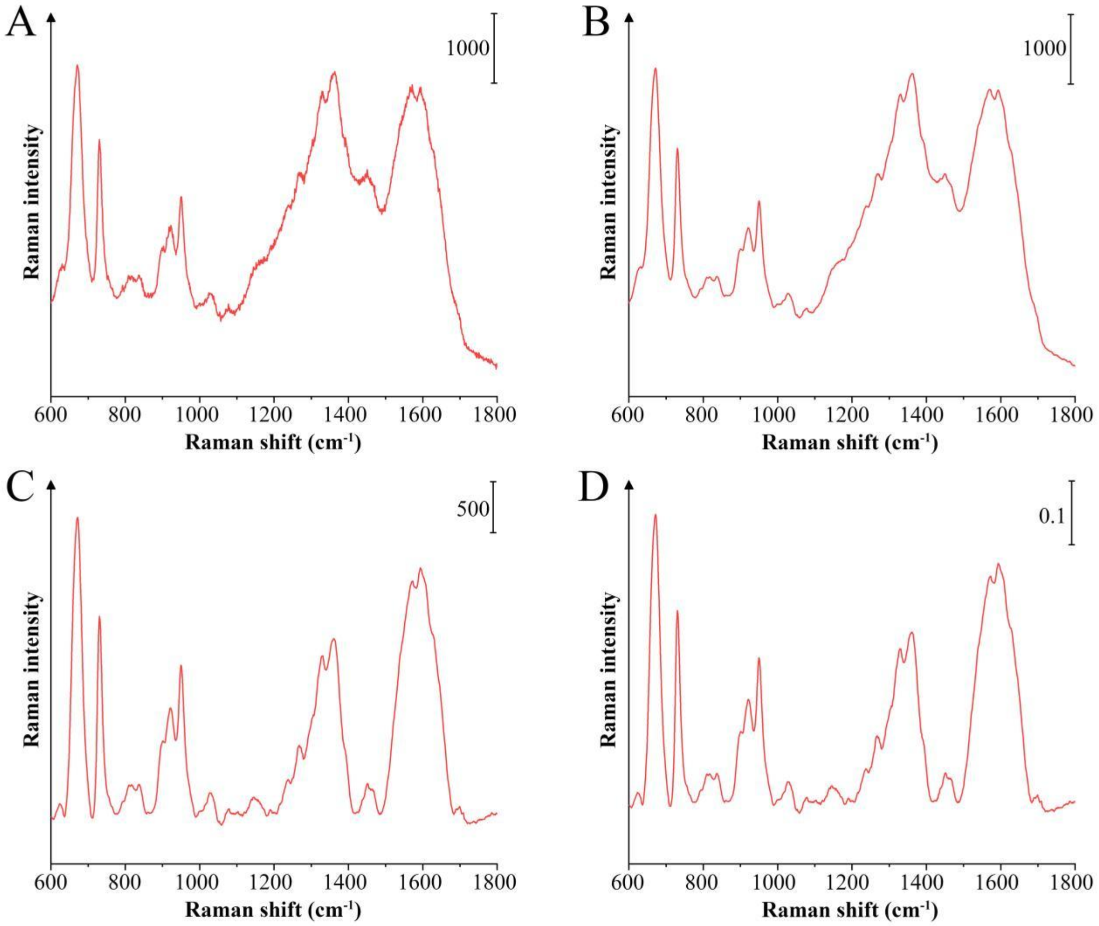

Figure S2. Representative spectra of each preprocessing step. (A) The original representative spectrum. (B) The representative spectrum after smoothing with a Savitzky-Golay filter. (C) The representative spectrum after baseline correction. (D) The representative spectrum after area normalization.

Construction of ML models on SERS spectra

We methodically executed four machine learning methods for the classification of Raman spectra: (1) A one-dimensional convolutional neural network (1D-CNN) that automates feature extraction via convolutional and pooling layers, followed by dense classification, optimized for high-dimensional spectral inputs; (2) Principal component analysis combined with linear discriminant analysis (PCA-LDA), which reduces dimensionality while preserving 95% of the variance and employs linear discriminant functions for clear class separation; (3) A support vector machine utilizing a radial basis function kernel (SVM), applying default gamma scaling to model nonlinear spectral boundaries; and (4) A random forest classifier consisting of 100 decision trees, employing Gini impurity for node splitting and bootstrap aggregation to capture nonlinear feature interactions. All models were trained with an 80/20 stratified train-test division and assessed according to accuracy, sensitivity, specificity, and AUC metrics.

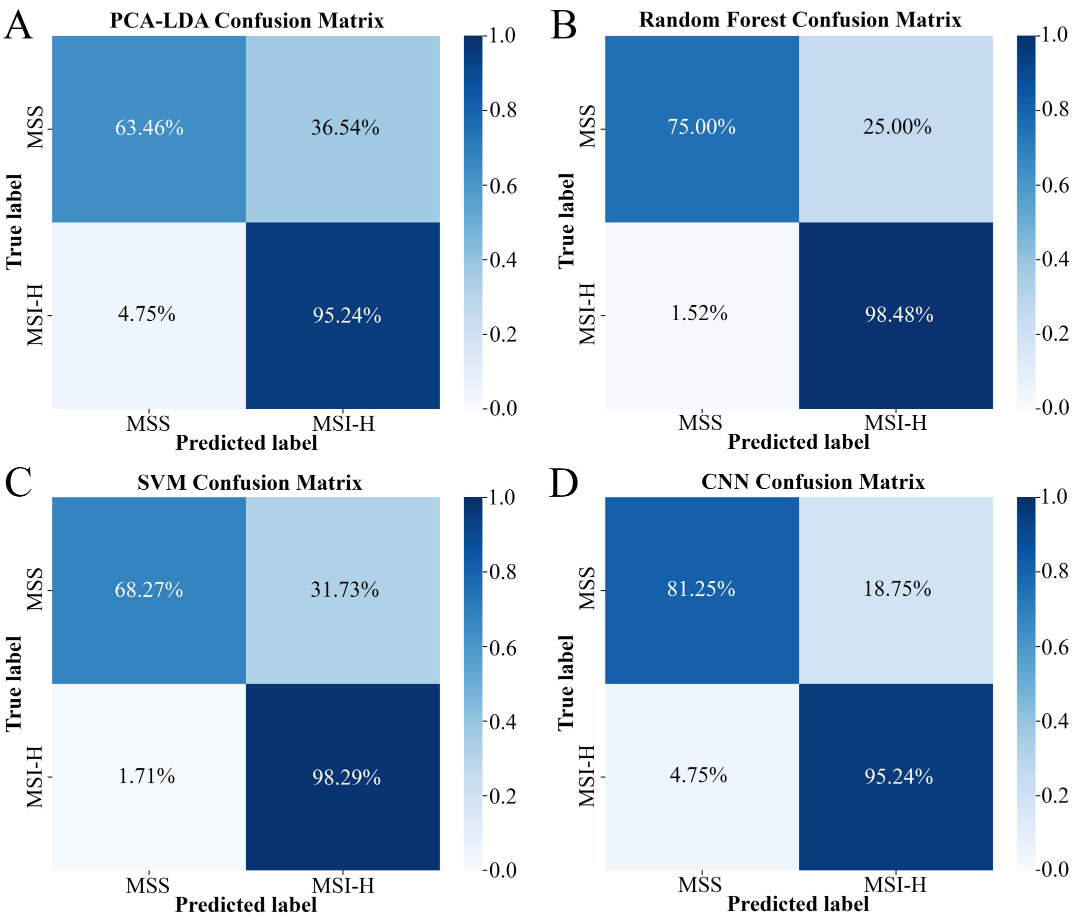

Figure S3. Confusion matrices demonstrating the classification efficacy of PCA-LDA, RF, SVM, and CNN in differentiating between the MSS and MSI-H subgroups.

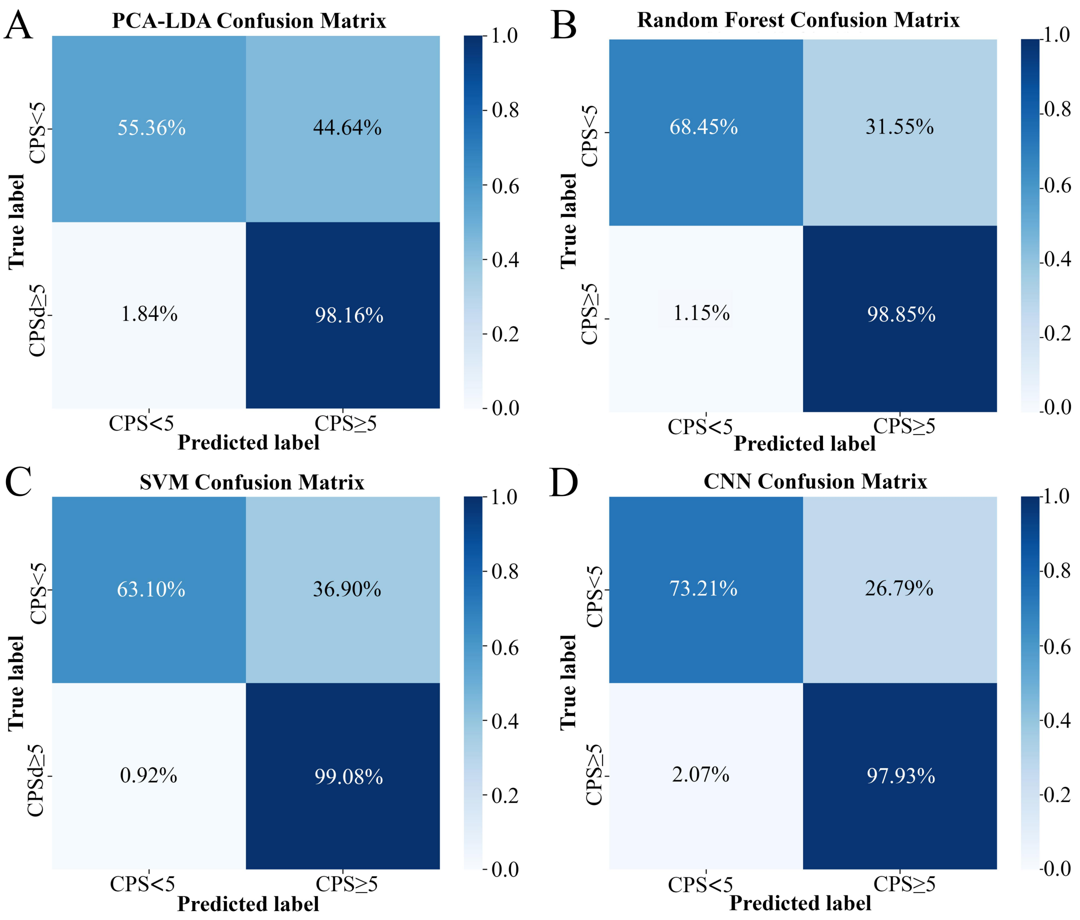

Figure S4. Confusion matrices demonstrating the classification efficacy of PCA-LDA, RF, SVM, and CNN in differentiating between the PD-L1(CPS<5) and PD-L1(CPS≥5) subgroups.

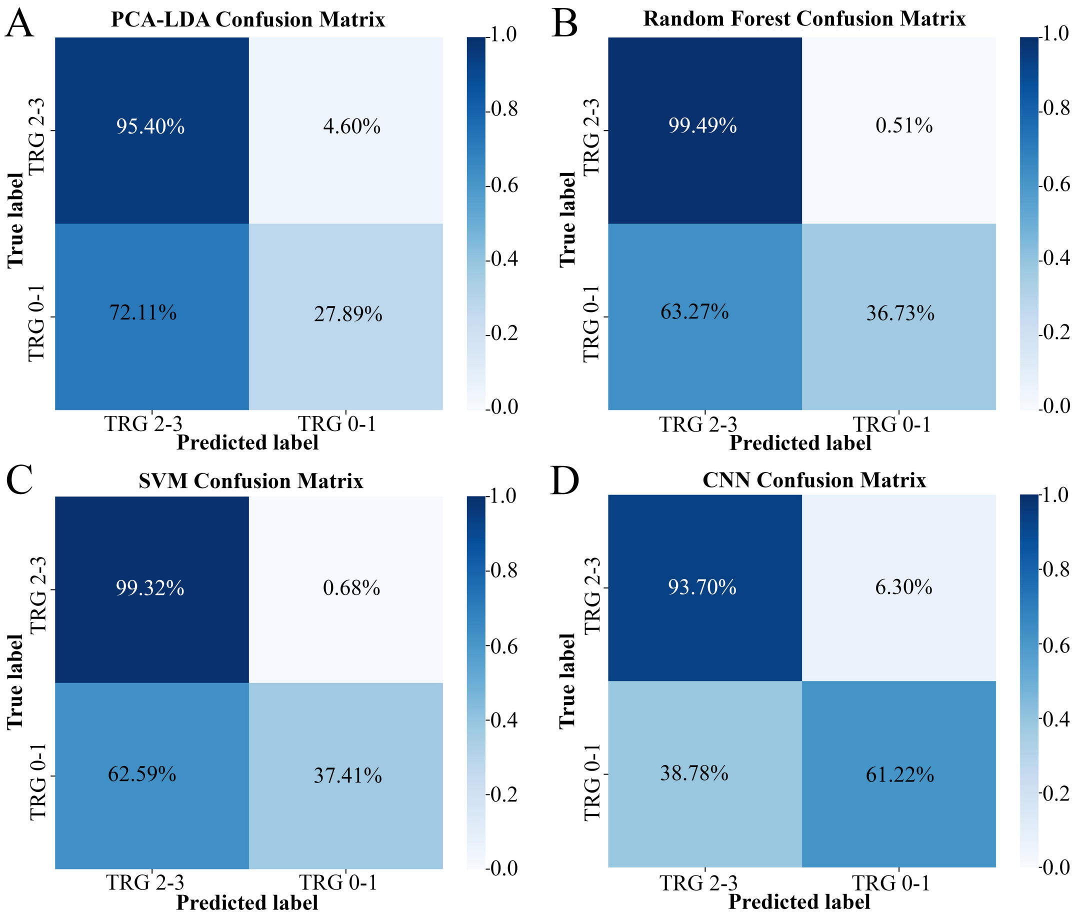

Figure S5. Confusion matrices demonstrating the classification efficacy of PCA-LDA, RF, SVM, and CNN in differentiating between the TRG 0-1 and TRG 2-3 subgroups.

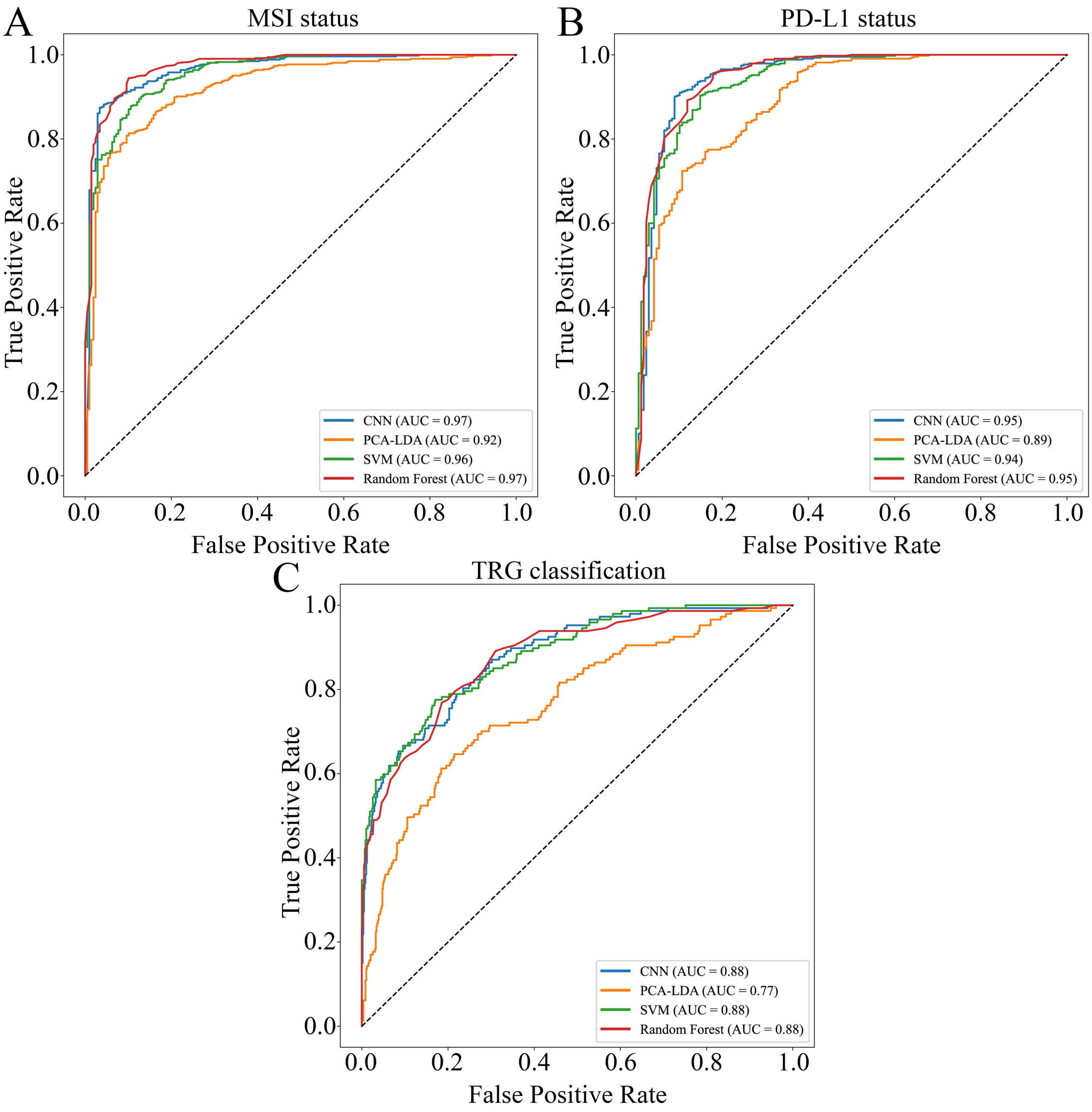

Figure S6. ROC curves of MSI status, PD-L1 status and TRG subgroups based on PCA-LDA, RF, SVM, and CNN.

Construction of Raman score

Partial least squares regression (PLSR) is a supervised dimension-reduction and calibration method that relates high-dimensional, strongly collinear predictors to an outcome by extracting components that maximize the predictor–outcome covariance.[4–7] In this study, PLSR projects the high-dimensional spectral matrix onto a reduced feature space spanned by latent variables—weighted linear combinations of the original wavenumber intensities that maximize covariance with the outcome. Two latent variables (PLS1 and PLS2) were selected by cross-validation to balance parsimony and prognostic performance. Each latent variable is accompanied by a loading (weight) profile that reflects the relative contribution of individual Raman bands to that component. For each case, the original spectrum was projected onto this latent space to obtain PLS1 and PLS2 scores. These scores were then used as covariates in a Cox proportional hazards model, and the model's linear predictor was defined as the Raman score,

$$\text{Raman score} = \beta_1 \text{PLS1} + \beta_2 \text{PLS2}$$

β_1 and β_2 represent the Cox model regression coefficients for PLS1 and PLS2, respectively. Note that Cox models do not incorporate an intercept term. The Raman score was assessed both on its own (univariable Cox) and in combination with clinical covariates (multivariable Cox). The Raman score maintained statistical significance in both models (refer to Tables 2 and 3 in the manuscript), highlighting its strong association with survival and corroborating the validity of our methodological approach.

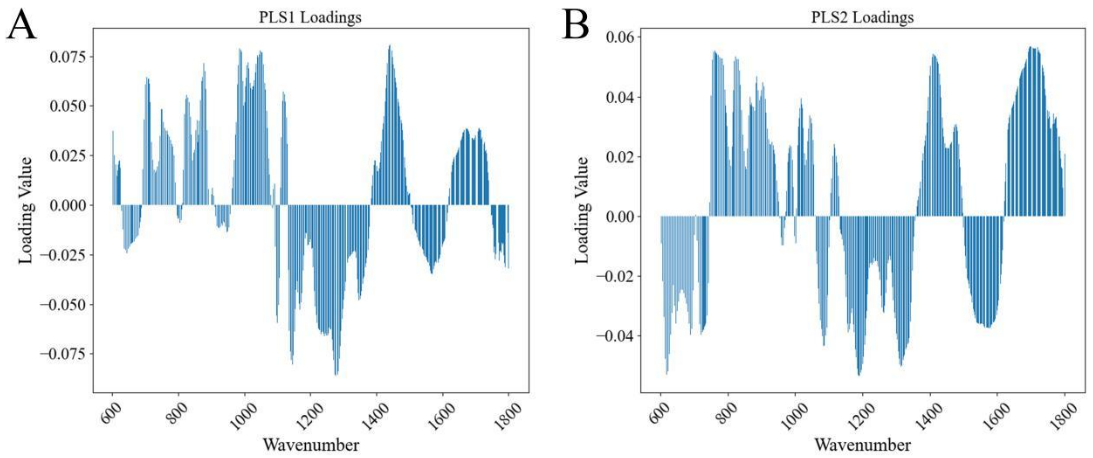

Figure S7. Loading plots of latent variables (PLS1 and PLS2).

Table S1. PLS scores of the Raman spectral features extracted by Partial Least Squares Regression.

Patient ID	PLS1 Score	PLS2 Score
1	1.887247084	-9.236220159
2	-1.311985764	-3.056010726
3	-8.012176753	-1.248607621
4	1.52221842	33.43251383
5	-4.602573025	-14.7656655
6	0.463291864	-4.787656821
7	-0.142653336	3.18990725
8	19.95825915	31.83421586
9	0.544833817	-15.2462586
10	-5.369798633	-8.886674706
11	11.01568972	-20.81912969
12	-3.360996307	3.253060267
13	-2.432358169	-10.20755743
14	-32.21282988	11.28352173
15	-4.122896073	8.938987874
16	3.515400919	-10.70849467
17	0.002500727	-10.10598726
18	0.323199461	2.136053792
19	0.104793227	-11.88758284
20	2.047045186	-7.34808396
21	4.104532368	-0.752214345
22	-4.396846364	17.76242874
23	17.10506678	-15.84826048
24	-6.809820928	8.772891007
25	-0.773956046	-8.748020521
26	9.885366908	-14.38985274
27	-5.0572552	0.419854805
28	-5.839815087	13.14919977
29	-6.350382757	-4.35677942
30	12.64552206	37.77509036
31	5.671376629	0.451332211

REFERENCES

1. Grosset A-A, Dallaire F, Nguyen T, Birlea M, Wong J, Daoust F, *et al.* Identification of intraductal carcinoma of the prostate on tissue specimens using Raman micro-spectroscopy: A diagnostic accuracy case–control study with multicohort validation. *PLOS Med* 2020;17:e1003281. doi: 10.1371/journal.pmed.1003281

2. Lee PC, Meisel D. Adsorption and surface-enhanced Raman of dyes on silver and gold sols. *J Phys Chem* 1982;86:3391–3395. doi: 10.1021/j100214a025

3. Bi X, Wang J, Xue B, He C, Liu F, Chen H, *et al.* SERSomes for metabolic phenotyping and prostate cancer diagnosis. *Cell Rep Med* 2024;5:101579. doi: 10.1016/j.xcrm.2024.101579

4. Mevik B-H, Wehrens R. The pl package: Principal component and partial least squares regression in R. *J Stat Softw* 2007;18:1–24.

5. Wold S, Sjöström M, Eriksson L. PLS-regression: a basic tool of chemometrics. *Chemom Intell Lab Syst* 2001;58:109–130. doi: 10.1016/S0169-7439(01)00155-1

6. Abdi H. Partial least squares regression and projection on latent structure regression (PLS Regression). *WIREs Comput Stat* 2010;2:97–106. doi: 10.1002/wics.51

7. Geladi P, Kowalski BR. Partial least-squares regression: a tutorial. *Anal Chim Acta* 1986;185:1–17. doi: 10.1016/0003-2670(86)80028-9
